# Pan-ebolavirus serology study of healthcare workers in the Mbandaka Health Region, Democratic Republic of the Congo

**DOI:** 10.1371/journal.pntd.0010167

**Published:** 2022-03-07

**Authors:** Kelly C. L. Shaffer, Sean Hui, Anna Bratcher, Liam B. King, Rachel Mutombe, Nathalie Kavira, Jean Paul Kompany, Merly Tambu, Kamy Musene, Patrick Mukadi, Placide Mbala, Adva Gadoth, Brandyn R. West, Benoit Kebela Ilunga, Didine Kaba, Jean Jacques Muyembe-Tanfum, Nicole A. Hoff, Anne W. Rimoin, Erica Ollmann Saphire

**Affiliations:** 1 Center for Infectious Disease and Vaccine Research, La Jolla Institute for Immunology, La Jolla, California, United States of America; 2 Department of Epidemiology, UCLA Fielding School of Public Health, University of California, Los Angeles, Los Angeles, California, United States of America; 3 Department of Medicine, University of California, San Diego, La Jolla, California, United States of America; 4 Department of Immunology and Microbiology, The Scripps Research Institute, La Jolla, California, United States of America; 5 Institut National de Recherche Biomedicale, Kinshasa, Democratic Republic of the Congo; 6 Directorate of Disease Control, Ministry of Public Health, Kinshasa, Democratic Republic of the Congo; 7 Kinshasa School of Public Health, Kinshasa, Democratic Republic of the Congo; Liverpool School of Tropical Medicine, UNITED KINGDOM

## Abstract

Although multiple antigenically distinct ebolavirus species can cause human disease, previous serosurveys focused on only *Zaire ebolavirus* (EBOV). Thus, the extent of reactivity or exposure to other ebolaviruses, and which sociodemographic factors are linked to this seroreactivity, are unclear. We conducted a serosurvey of 539 healthcare workers (HCW) in Mbandaka, Democratic Republic of the Congo, using ELISA-based analysis of serum IgG against EBOV, *Sudan ebolavirus* (SUDV) and *Bundibugyo ebolavirus* (BDBV) glycoproteins (GP). We compared seroreactivity to risk factors for viral exposure using univariate and multivariable logistic regression. Seroreactivity against different GPs ranged from 2.2–4.6%. Samples from six individuals reacted to all three species of ebolavirus and 27 samples showed a species-specific IgG response. We find that community health volunteers are more likely to be seroreactive against each antigen than nurses, and in general, that HCWs with indirect patient contact have higher anti-EBOV GP IgG levels than those with direct contact. Seroreactivity against ebolavirus GP may be associated with positions that offer less occupational training and access to PPE. Those individuals with broadly reactive responses may have had multiple ebolavirus exposures or developed cross-reactive antibodies. In contrast, those individuals with species-specific BDBV or SUDV GP seroreactivity may have been exposed to an ebolavirus not previously known to circulate in the region.

## Author summary

*Zaire ebolavirus* is known to circulate in the Mbandaka region of the Democratic Republic of the Congo, causing outbreaks in 2018 and 2020. However, we do not know the range of exposure to the local population. Here, we examined the seroprevalence of 539 local Congolese healthcare workers in the Mbandaka region with no known ebolavirus exposure. We found serological evidence indicating contact with at least one species of ebolavirus from these donors. Seroreactivity among the donors to the different glycoprotein antigens ranged between 2.2–4.6%. We observed correlations between jobs with indirect access to patients and a higher seroprevalence, which may be due to less training and less access to personal protective equipment. Our findings suggest that exposure to ebolaviruses may be more frequent than previously known and that lesser-skilled individuals in healthcare work may have a higher likelihood of ebolavirus exposure.

## Introduction

There are six known, antigenically-distinct *Ebolavirus* species: Ebola (Zaire; EBOV), Bundibugyo (BDBV), Sudan (SUDV), Taï Forest (TAFV), Reston (RESTV), and Bombali viruses (BOMV), with the first four known to cause severe disease in humans [1]. Ebolaviruses display a trimeric glycoprotein (GP) on the viral surface that is a main target of the host humoral immune response. GP contains a heavily glycosylated glycan cap and mucin-like domain (MLD), which shield the receptor binding site and must be removed to facilitate infection [2].

As the etiological agent of Ebola Virus Disease (EVD), ebolaviruses have caused over two-dozen outbreaks throughout Africa since 1976, with case-fatality rates ranging from 25 to 90% [3]. EVD presents a special challenge in diagnosis as its prodrome mirrors that of bacterial infections and commonly circulating diseases such as malaria. Clinical signs and symptoms are non-specific and typically include fever, vomiting, diarrhea, musculoskeletal pain, headache, and sometimes hemorrhage [4–6]. Undetected cases and evidence of possible asymptomatic and paucisymptomatic infections can further complicate diagnosis [7,8]. Further, EBOV has been documented to re-emerge from survivors, spread to their sexual partners and introduce new chains of transmission several years after the original survivors’ initial clinical course [9]. Asymptomatic infection and re-emergence make understanding population seroprevalence a high priority.

**Fig 1:**
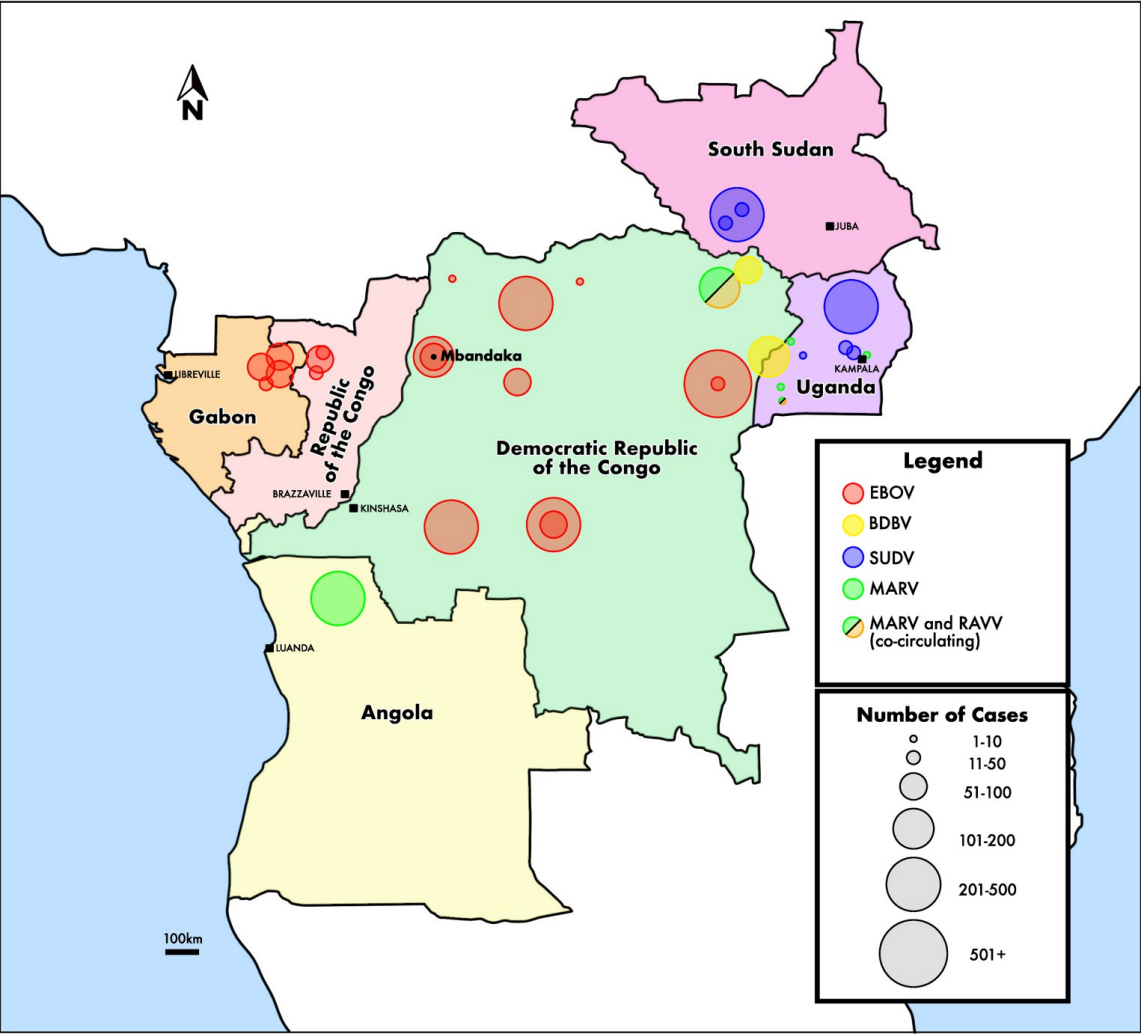
Filovirus Outbreaks in Central Africa. Mbandaka was the site of two EBOV outbreaks: 2018 (54 cases) and 2020 (130 cases). Other outbreaks in the northwestern DRC occurred in: Yambuku (1976; 318 cases; 540 km from Mbandaka), Tandala (1977; 1 case; 350 km), Boende (2014; 69 cases; 300 km) and Likati (2017; 8 cases; 725 km from Mbandaka). The surrounding countries of Angola and Uganda experienced outbreaks of marburgviruses while Gabon, Republic of the Congo, Uganda and present-day South Sudan have experienced numerous outbreaks of multiple ebolaviruses [3]. Within the DRC, most confirmed outbreaks have been due to EBOV infections. In two instances, cases of MARV and RAVV were documented simultaneously, and numerous filoviruses likely co-circulate due to their close geography [6,10]. Map adapted from USGS. (https://ngmdb.usgs.gov/topoview/viewer/#5/-0.931/21.904). Abbreviations: EBOV = *Zaire ebolavirus*, BDBV = *Bundibugyo ebolavirus*, SUDV = *Sudan ebolavirus*, MARV = *Marburg marburgvirus*, RAVV = *Ravn Marburgvirus*.

The increasing frequency of ebolavirus emergence in affected areas underscores the importance of serological surveys. The Democratic Republic of the Congo (DRC) has had 12 confirmed EVD outbreaks at the time of writing, with at least one occurring every year since 2017 (Fig 1) [3,10]. The northwestern region of the DRC has experienced six documented EVD outbreaks, including recent episodes in the Équateur province in 2018 and 2020. No other ebolaviruses have ever been known to circulate in this region. Despite being a center of ebolavirus activity, seroprevalence among citizens of northwestern DRC has been understudied. Serology to explore seroreactivity to the variety of ebolaviruses can help further our understanding of their distribution and virulence.

In serology, the quality, folding, and oligomeric state, as well as the viral species of the antigen used, are of key importance to detection of antibodies [11–13]. Specificity and accuracy of serology assays are improved by the use of antigens with conformations and oligomeric states which are representative of those displayed on authentic virus, rather than poorly folded proteins or separated monomers. Furthermore, inclusion or deletion of flexible, highly glycosylated, and masking domains of the antigen influence antibody detection [14]. In most prior studies on Ebola virus serology, complete GP ectodomain (EBOV GPe) was used. GPe contains the mucin-like domain (MLD), which allows for detection of antibodies against epitopes in the MLD itself, but may limit detection of antibodies against other surfaces of the GP core if shielded by the MLD [14]. We sought to compare MLD-containing and MLD-deleted GP antigens in detection.

Most previous serological studies in the DRC examined the prevalence of antibodies in known EVD survivors or close contacts of EVD patients, and few studies have been performed in the northwestern part of the country [15,16]. Two studies analyzed more general populations in northwestern DRC: Lucas et al. studied 19 market workers in Mbandaka, and revealed one individual seroreactive to EBOV GPe, but not to other ebolavirus GPs [17]. Doshi et al. analyzed 582 health care workers (HCWs) who worked through the 2014 Boende EBOV outbreak, and detected 22.7% seroreactivity to EBOV GPe, but did not test other ebolavirus antigens [18].

In this analysis, we used structurally well-characterized antigens to evaluate serum anti-GP IgG reactivity of 539 HCWs in the Mbandaka, Wangata, and Bolenge Health Zones of the Équateur province against three ebolaviruses. Of the HCWs surveyed, 525 had never knowingly taken care of or been exposed to an EVD patient and only 12 reported a known exposure to someone with EVD. Additionally, we paired seroreactivity results with sociodemographic data to determine which activities and demographic characteristics may be associated with potential viral exposure in the workplace or in the community. Finally, by testing for antibodies against multiple species of ebolaviruses, this study allows for a more comprehensive view of potential ebolavirus cross-reactivity observed in HCWs in northwestern DRC.

## Materials and methods

### Ethics statement

This study was approved by Institutional Review Boards (IRBs) at the University of Kinshasa in Kinshasa, DRC (ESP/CE/022/2017) and at the University of California, Los Angeles (UCLA) (IRB#16–001346 and IRB#20–00029). Additionally, the study was approved by the Scientific Committee for Ebola Research during an outbreak at the National Institute of Biomedical Research (INRB), under the Ministry of Health. Before any study-related procedures were conducted, participants signed or marked the approved informed consent form.

### Sample population and study design

Study participants were enrolled between June and July 2018, during the May to July 2018 outbreak of EVD in the region. Three semi-urban health zones of Mbandaka city were targeted: Mbandaka, Wangata, and Bolenge, which are all in Équateur province, DRC. In the three health zones, 27 randomly selected health facilities were contacted and asked to participate in the study. The administration of each participating facility provided a list of their HCWs. Based on the WHO definition of additional individuals who may be involved in healthcare work, a listing of traditional healers or pastors working in proximity to participating facilities was also obtained from the selected facilities who work with them [19,20]. Among those identified in these lists, individuals were eligible for the study if they were at least 18 years old, had no fever (<38°C), and had no known health issues or other self-reported acute illness at the time of enrollment. A total of 544 individuals completed an informed consent process and were enrolled in the study. Of these, 539 were included in this analysis; the remaining five participants had insufficient sociodemographic survey data.

### Study procedures

After obtaining informed consent, participants completed an electronic questionnaire using an Open Data Kit (ODK) Collect survey administered by trained interviewers. Surveys were conducted in the participant’s preferred local language (French or Lingala), and data on sociodemographic and behavioral characteristics were collected. Data concerning potential exposures to EVD using previously defined criteria to describe direct, indirect and unlikely contact were also collected [18].

Trained phlebotomists collected blood samples using venipuncture methods. Whole blood samples were collected in red-top tubes (BD) for serum isolation. Collected samples were processed in the field. Briefly, samples were centrifuged before aliquoting into cryotubes and heat inactivation (56°C for 30 minutes). Samples were then stored at -20°C for shipment to the National Institute for Biomedical Research in Kinshasa, DRC where they were stored at -80°C before testing onsite.

### Protein expression and purification

Soluble ectodomain, lacking the C-terminal transmembrane domain, of ebolavirus surface glycoproteins (GPs) were produced by stable expression in *Drosophila melanogaster* S2 cells as described elsewhere [21]. Briefly, Effectene (Qiagen) was used to transfect S2 cells with a modified pMT-puro vector plasmid containing the GP gene of interest, followed by stable selection of transfected cells with 6μg/mL puromycin. Cells were cultured at 27°C in complete Schneider’s medium for selection and then adapted to Insect Xpress medium (Lonza) for large-scale expression in 2L Erlenmeyer flasks. Expression of secreted GP was induced with 0.5mM CuSO_4_, and supernatants were harvested after 5 days. All GPs have a C-terminal double Strep tag and were purified using 5mL Strep-trap HP columns (GE). Proteins were then diluted using 10mM Tris-buffered saline (Tris-HCl, pH 7.5, 150mM NaCl [TBS]). SUDV GP has a T4 fibritin trimerization domain to facilitate formation of native quaternary structure.

### Multi-antigen ELISAs

For serological assays used to analyze immunoglobulin G (IgG) seroprevalence, MLD-containing (EBOV GPe) and MLD-deleted EBOV Mayinga GP (EBOV GPΔMuc), MLD-deleted BDBV GP (BDBV GPΔMuc), and SUDV Boniface GP (SUDV GPΔMuc) were used. The three-dimensional structure for each antigen was confirmed by cryoEM or X-ray crystallography [12,13, 22–24]. High-binding, 96 half-well ELISA plates (Corning) were coated with 50μl of the indicated GP antigen at 2μg/mL in 0.1M carbonate buffer pH 8.5 for one hour at room temperature. After washing with 0.05% Tween-20 in phosphate buffered saline (PBS), the wells were blocked overnight at 4°C with PBS containing 3% casein, followed by an additional wash step. Human sera diluted 500-fold in PBS with 3% casein was added in duplicate wells and incubated for 30 minutes at room temperature. Horseradish-peroxidase conjugated anti-human IgG Fc secondary antibody (Thermo Fisher Scientific) diluted 1:10,000 in PBS with 3% casein was then allowed to bind for one hour at room temperature. Ultra-TMB (3,3′,5,5′-tetramethylbenzidine) substrate (Thermo Fisher Scientific) was added and incubated for 4 minutes before the reaction was quenched with 1M H_2_SO_4_. Optical density was measured at 450nm. Non-immune pooled sera from African individuals served as a control for binding specificity (Zalgen Labs). Adimab-15878 IgG, a pan-ebolavirus monoclonal antibody isolated from an EVD survivor [12,25], was used as a positive control at 1μg/mL and a five-point, five-fold serial dilution was performed. An anti-Strep tag antibody (Sigma-Aldrich) diluted 1:5000 in PBS with 3% casein served as a secondary antigen control.

### Statistical analysis

**Fig 2:**
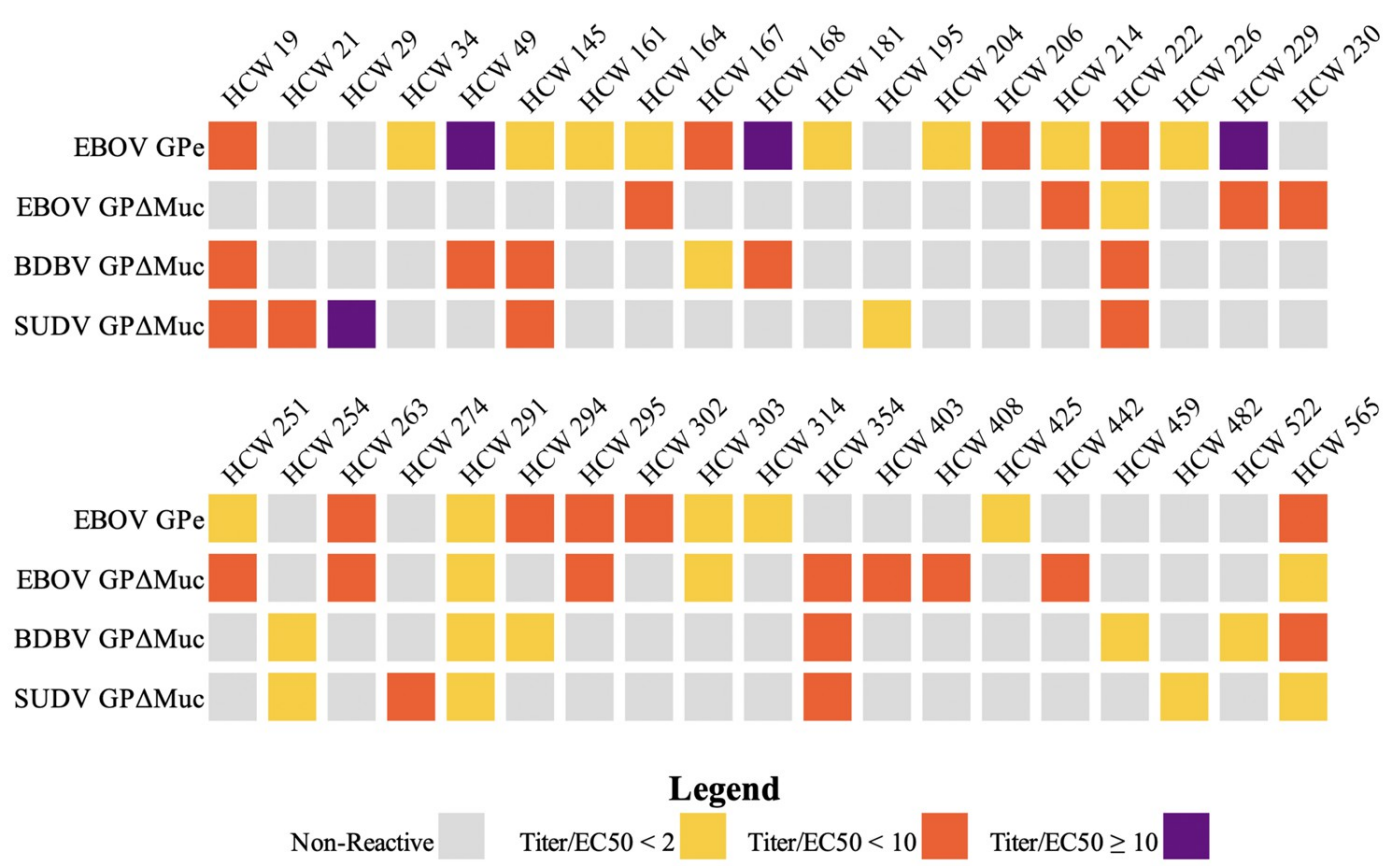
Patient Seroreactivity by Antigen. The breadth of participant seroreactivity against ebolaviruses in the 36 reactive samples is illustrated. None of these individuals reported exposure to confirmed or probable EVD cases. The samples were characterized by the interpolation of the IgG concentration (Titer) divided by the EC_50_ of Adimab-15878 for the plate. They were further classified into separate groups by the level of the Titer/EC_50_ ratio: 1<2 for Weak Reactivity, 2≤10 for Moderate Reactivity, and 10+ for Strong Reactivity. Four samples were strongly reactive: 3 EBOV GPe (Titer/EC_50_: 86 (HCW 49), 16 (HCW 168), 16 (HCW 229)) and 1 SUDV GPΔMuc (Titer/EC_50_: 30 (HCW 29)).

Descriptive statistics for sociodemographic and other characteristics were tabulated. Optical density measures were transformed to antibody titers using linear regression coefficients based on the standard curve for Adimab-15878 for each plate. The geometric mean of antibody titer and proportion of samples having an elevated baseline titer were obtained for the four GP antigens. A sample was considered to have an elevated antibody titer if the average of its duplicate OD 450nm values was higher than the EC_50_ value of Adimab-15878 for the corresponding standard curve for each plate to account for interplate variation. Adimab-15878 IgG EC_50_ values were calculated with Prism 9.0 software using a dose-response four-parameter nonlinear regression. Samples that were 1 to 2-fold, 2 to 10-fold, and 10-fold higher than the Adimab-15878 EC_50_ were classified as weakly, moderately, and strongly reactive, respectively (Fig 2).

Next, intraclass correlation coefficients (ICCs) comparing the log of antibody titers were obtained for each pair of titers. Unadjusted relationships between each ebolavirus titer and demographic or possible EVD exposures were then obtained using univariate logistic regression. Multivariable analysis was not completed due to sparse data. For ICCs, a 95% confidence interval (CI) is provided and a 95% CI that did not cross the null value of 0 was considered to be evidence of a correlation between antigens. For odds ratios, 95% CIs that did not contain 1 were considered to show evidence of an association. No corrections were made for multiple comparisons. R software was used for all statistical analyses except EC_50_ calculations [26].

## Results

Among the 539 HCWs enrolled, the median age was 42 years old [interquartile range (IQR): 34, 53] and 55.7% were between 30 and 49 years-old. Around half (51.8%) were female, 71.2% were married or cohabitating, and 58.5% had college, university or graduate school education (Table 1). Nurses comprised 54.8% of the survey population, and the remaining professions are listed in Table 1. A total of 58% of participants were classified as having direct contact with any patients in their current position and 28.3% were classified as having indirect contact with patients. Only 2.2% reported contact with a confirmed, probable or suspected EVD case. One participant was uncertain about their EVD exposure history.

**Table 1:** Sample characteristics of 539 participants from Mbandaka and the surrounding areas in the Democratic Republic of the Congo, August 2018.

	Median	Q1, Q3
Age	42	34, 53
	Frequency (n)	Percent (%)
Sex		
*Male*	260	48.2
*Female*	279	51.8
Age^a^		
*18–29*	62	11.7
*30–39*	163	30.7
*40–49*	133	25.0
*50–59*	108	20.3
*60–82*	65	12.2
Education^b^		
*None*	6	1.2
*Any primary school or apprenticeship*	110	22.5
*Finished secondary school*	87	17.8
*College/University or Graduate school*	286	58.5
Marital status		
*Single*	91	16.9
*Married or living together as married*	384	71.2
*Divorced*, *separated*, *or widowed*	64	11.9
Type of Healthcare worker^c^		
*Nurse*	295	54.8
*Physician*	1	0.2
*Supervisor*	2	0.4
*Health communication officer*	3	0.6
*Laboratory staff*	18	3.3
*Administrator*	39	7.2
*Room attendant*	45	8.4
*Hygienic service*	50	9.3
*Traditional healer/pastor*	1	0.2
*Medical/nurse student*	5	0.9
*Birth attendant*	10	1.9
*Maintenance*	17	3.2
*Community volunteer*	30	5.6
*Pharmacist*	6	1.1
*Other*	16	3.0
Contact with patients^c^		
*Direct*	312	58.0
*Indirect*	152	28.3
*No contact*	74	13.8
Has ever had contact with a confirmed, probable, or suspected EVD case?^d^		
*Yes*	12	2.2
*No*	526	97.8

a. 8 missing

b. 50 missing

c. 1 missing

d. 1 don’t know/refused

Overall, seroreactivity of HCWs against the four ebolavirus GPs ranged between 2.2 and 4.6%, depending on the antigen (Table 2). None of the 12 individuals with self-reported exposure to a confirmed, probable or suspected EVD case demonstrated seroreactivity to an ebolavirus antigen.

**Table 2:** Ebola virus-specific and ebolavirus cross-reactive antibody levels among 539 participants from Mbandaka and the surrounding areas in the Democratic Republic of the Congo, August 2018.

	Geometric mean titer concentration (95% CI)	Number with elevated baseline titer (%)
EBOV GPe	42.3 (38.4, 46.5)	25 (4.6)
EBOV GPΔMuc	20.9 (19.3, 22.6)	15 (2.8)
BDBV GPΔMuc	21.3 (20.2, 22.5)	13 (2.4)
SUDV GPΔMuc	16.5 (15.6, 17.6)	12 (2.2)

Two EBOV GP constructs were used for analysis: 1) MLD-containing (GPe), and 2) MLD-deleted (GPΔMuc). Interestingly, 25 (4.6%) samples reacted with EBOV GPe, but only 15 (2.8%) reacted with EBOV GPΔMuc. Ten (1.9%) reacted with both EBOV GP constructs. Among those seroreactive against EBOV GPe, three (12%) participants showed strong, nine (36%) medium, and 13 (52%) weak IgG responses (Fig 2). Medium and weak reactivity against EBOV GPΔMuc was seen for ten (67%) and five (33%) individuals, respectively. No strong responses were seen against EBOV GPΔMuc.

Multiple individuals reacted with more than one ebolavirus. Six individuals showed an IgG response against all three viruses, and three of those individuals responded to all four antigens. Of the 13 individuals in the study who were seroreactive against BDBV GP, ten reacted with both EBOV and BDBV GPs, one reacted with both SUDV and BDBV, and two reacted only with BDBV GP. Seroreactivity against SUDV GP was observed in 12 individuals; six of whom reacted with EBOV and SUDV GPs, one reacted with BDBV and SUDV GPs, and five reacted with SUDV GP only.

Weak correlation existed between reactivity to EBOV GPΔMuc and BDBV GPΔMuc (Fig 2 and Table 3). Additionally, there was very weak correlation between reactivity to SUDV GPΔMuc and to BDBV GPΔMuc. No other pairwise relationships were statistically significant.

**Table 3:** Intraclass correlation coefficients (ICCs) and 95% confidence intervals for pairwise concurrence of Ebola virus-specific and ebolavirus cross-reactive antibody levels among of 539 participants from Mbandaka and the surrounding areas in the Democratic Republic of the Congo, August 2018.

	EBOV GPe	EBOV GPΔMuc	BDBV GPΔMuc	SUDV GPΔMuc
EBOV GPe	1	0.01 (-0.08, 0.09)	0.06 (-0.02, 0.15)	0.00 (-0.08, 0.09)
EBOV GPΔMuc		1	0.26 (0.18, 0.34)	0.06 (-0.03, 0.14)
BDBV GPΔMuc			1	0.09 (0.01, 0.17)
SUDV GPΔMuc				1

Sex was associated with seroreactivity in our univariate analysis, with reduced likelihood of EBOV GPe and BDBV GPΔMuc seroreactivity among female participants compared to male participants (Table 4). Seroreactivity was also associated with certain types of healthcare work. Laboratory staff were around nine times more likely to be seroreactive for EBOV GPe than nurses (OR 9.08, 95% CI: 2.49, 33.12), and birth attendants were nearly 11 times more likely to be seroreactive for EBOV GPΔMuc than nurses (OR 10.81, 95% CI: 1.02, 114.36). Additionally, community health volunteers were more likely to be seroreactive against each of the four antigens compared to nurses. In terms of patient contact, those having indirect contact were more likely to have antibodies against each EBOV GPe and EBOV GPΔMuc relative to those having direct patient contact.

**Table 4:** Predictors of Ebola virus-specific and ebolavirus cross-reactive antibody levels of 539 participants from Mbandaka and the surrounding areas in the Democratic Republic of the Congo, August 2018.

	EBOV GPe		EBOV GPΔMuc		BDBV GPΔMuc		SUDV GPΔMuc	
Predictor*	Odds Ratio	95% CI	Odds Ratio	95% CI	Odds Ratio	95% CI	Odds Ratio	95% CI
Sex								
*Male*	reference		reference		reference		reference	
*Female*	0.22	0.08, 0.59	0.46	0.15, 1.35	0.16	0.04, 0.74	0.66	0.21, 2.10
Age^a^								
*18–29*	reference		reference		reference		reference	
*30–39*	0.55	0.19, 1.60	1.15	0.23, 5.84	2.74	0.33, 22.72	1.53	0.17, 14.00
*40–49*	0.36	0.11, 1.24	0.93	0.17, 5.22	0.93	0.08, 10.47	1.89	0.21, 17.29
*50–59*	0.18	0.03, 0.90	0.28	0.02, 3.16	1.74	0.18, 17.13	1.74	0.18, 17.13
*60–82*	0.45	0.11, 1.89	0.95	0.13, 6.98				
Education								
*Any primary school or apprenticeship*	reference		reference		reference		reference	
*Finished secondary school*	1.24	0.34, 4.51	0.92	0.16, 5.11	5.68	0.58, 55.51		
*College/University or Graduate school*	1.42	0.54, 3.74	1.27	0.38, 4.18	5.17	0.65, 41.17		
Marital status								
*Single*	reference		reference		reference		reference	
*Married or living together as married*	0.59	0.24, 1.46	0.95	0.26, 3.42	0.46	0.14, 1.57	1.19	0.26, 5.53
*Divorced*, *separated*, *or widowed*					0.35	0.04, 3.16		
Type of Healthcare worker								
*Nurse*	reference		reference		reference		reference	
*Laboratory staff*	9.08	2.49, 33.12	5.73	0.57, 57.99	2.83	0.32, 24.89	2.42	0.28, 20.82
*Administrator*	1.82	0.38, 8.75	2.70	0.27, 26.68	1.34	0.16, 11.43		
*Room attendant*			4.53	0.74, 27.88			0.94	0.11, 7.78
*Hygienic service*	2.76	0.82, 9.34	4.06	0.66, 24.91				
*Birth attendant*			10.81	1.02, 114.36				
*Maintenance*					3.44	0.39, 30.56		
*Community volunteer*	6.36	1.98, 20.42	14.97	3.18, 70.53	5.35	1.27, 22.61	4.57	1.12, 18.71
*Pharmacist*					5.35	0.58, 49.20		
Contact with patients								
*Direct*	reference		reference		reference		reference	
*Indirect*	3.34	1.39, 8.02	5.14	1.56, 16.98	1.46	0.41, 5.25	1.57	0.49, 5.03
*No contact*	1.05	0.22, 4.95	1.18	0.13, 10.70	2.41	0.59, 9.90		

*Due to low numbers in the study population, estimates could not be calculated for: No Education; Physician, supervisor, health communications officer, traditional healer/pastor, medical/nursing student, and other healthcare worker types; EVD exposure history

## Discussion

This study involved HCWs, most of whom (525/539; 97%) had never knowingly been in contact with EVD patients prior to sample collection in 2018. Despite an absence of confirmed EVD contact among 525 members of this cohort, we detected IgG responses among them to a variety of ebolaviruses, including some strong responses. These results suggest that a small portion of the HCWs analyzed may have unknowingly been exposed to an ebolavirus in their lifetime, either in the workplace or in the community. Furthermore, some individuals have generated cross-reactive antibodies to multiple ebolaviruses. Together with genetic sequencing showing animal-to-human spillover during the two EVD outbreaks with cases in Mbandaka city, our results suggest that ebolaviruses are endemic to this region of DRC [27–29].

In our cohort, male sex, age between 50–59 years-old, and certain types of healthcare work were associated with seroreactivity to at least one of the tested antigens. HCWs having indirect patient contact were also more likely to be seroreactive to EBOV than those with direct contact. We hypothesize that this difference could be associated with distribution and use of personal protective equipment (PPE) among HCWs. In resource-poor settings, PPE may only be provided to those with direct patient contact. Additionally, HCWs with direct patient contact may be more likely to be trained in infection control practices. However, infectious ebolaviruses can exist for hours on dry surfaces and for days in bodily fluids at room temperature, leaving those who come in contact with fluids or contaminated equipment susceptible [30]. This rationale could also explain why laboratory workers had a higher incidence of IgG response against EBOV GPe. Unfortunately, the limited size of our study cohort did not allow us to confirm whether other factors, such as living in close contact with potential virus reservoirs such as bats or non-human primates, contributed significantly to this association.

Community health volunteers, who go into rural villages to check the general health of their populations, were more likely to have antibodies against each of the four tested ebolavirus antigens compared to nurses in Mbandaka. Birth attendants were also more likely to be reactive for EBOV GPΔMuc, the only antigen for which a result was available because of sparse data. Birth attendants are exposed to body fluids, may not have routine access to PPE, and may rarely receive training on disease prevention methods, thus leaving them at high risk of contracting infectious diseases. Additional studies using larger cohorts are needed to fully examine the relationship of seroreactivity with PPE use, training on disease prevention, and/or exposure to ebolaviruses.

Notably, reactivity to one ebolavirus GP showed little correlation with reactivity to another: EBOV GPΔMuc and BDBV GPΔMuc showed only weak correlation, and BDBV GPΔMuc and SUDV GPΔMuc showed very weak correlation (Table 3). The observed EBOV-BDBV dual reactivity may simply be due to cross-reactivity: EBOV and BDBV are the most similar in the genus, sharing 77.3% of their genome overall and 65.2% of their GP amino acid sequence [31]. EBOV-SUDV cross-reactivity is somewhat less likely, as the amino acid sequences of EBOV and SUDV GPs are only 54.7% identical [32,33]. Meanwhile, BDBV and SUDV GP have 56.1% identical amino acid sequences [31,34,35]. The conserved portions of amino acid sequences across ebolavirus GPs are concentrated in the core of the protein, whereas amino acids on the surface against which antibodies might be directed are much more likely to be variable [36].

It is also notable that although EBOV is the only virus known to have circulated in the area, five people showed an IgG response to SUDV GP alone. This reactivity might be due to the greater trimeric stability of the SUDV antigen used. However, the lack of any reactivity to EBOV GPe, EBOV GPΔMuc or BDBV GP suggests an alternate possibility: that these individuals were exposed to SUDV or a SUDV-like virus (Fig 2: HCWs 21, 29, 195, 274 and 482), either in the Équateur region, or perhaps through travel to areas with known SUDV circulation. Notably, one of these five SUDV-only individuals (HCW 29) has a very strong SUDV-specific response (Titer/EC_50_ ratio: 30), and reported never living outside of Équateur Province.

Six of 38 seroreactive individuals reacted with all three ebolaviruses tested. Whether this broad reactivity results from multiple exposures, or simply cross-reactive antibodies that resulted from a single exposure or repeated exposures to a single virus is unclear. The scope of this study did not allow us to definitively determine if pan-ebolavirus or species-specific antibodies are associated with the seroreactivity to the various antigens. In general, these viruses are thought to be antigenically distinct, but monoclonal antibodies (mAbs) have been isolated from survivors that do target pan-ebolavirus epitopes [25,37,38]. Some of these mAbs were identified in Americans who were infected with only EBOV, suggesting that infection with a single species of ebolavirus can elicit a pan-ebolavirus IgG response [38]. It is likely that the antibodies responsible for this cross-reactivity bind to conserved epitopes amongst ebolaviruses, and thus could be elicited after a single exposure to a single type of ebolavirus.

Samples from the study cohort were only available after the outbreaks of EBOV, and as such, no direct comparison could be made with the study cohort itself or an additional, comparable cohort at an earlier time point to determine pre-outbreak seroprevalence. However, we believe that our negative control of pooled, non-immune sera from African individuals offers a relevant baseline to determine background seroreactivity. Further, a seroreactivity rate of between 2.2–4.6% is within the range of previously reported studies conducted on other assays [39].

In addition to providing epidemiologic findings, our study offers a novel ebolavirus assay. Our assay was initially developed to screen samples for use in antibody therapeutics, but its use can be expanded to research studies similar to this analysis. Compared to other serologic studies, including the Filovirus Animal Non-Clinical Group (FANG) and commercial Alpha Diagnostic International (ADI) anti-IgG ELISAs, our assay has a shorter incubation period (30 minutes instead of one hour), is performed at room temperature instead of 37°C, and uses a single-point high dilution of sera (one point at 1:500, instead of six-points from 1:50 to 1:1600 (FANG) or a single 1:250 dilution (ADI)) [40]. Further, this assay uses trimeric, pre-fusion GPs having confirmed folding that may better represent the viral surface GP than less well-characterized proteins, which are often monomeric. Ebolavirus GPs tend to separate into monomers, especially when expressed in HEK293 cells, a more common expression platform than the S2 cells used here [41]. Separation of the trimer into monomers exposes internal surfaces that may lead to non-specific binding and loss of quaternary epitopes. Together, the shorter incubation, lower temperature, higher sera dilution, and differences in GP structure mean that this assay likely favors binding of very high affinity antibodies, and thus may have higher specificity than those of previous studies. However, this specificity may miss individuals with weaker antibody responses and may underestimate the actual seroreactivity of the study population. The identification of individuals with strong responses is thus striking.

We compared serological responses to EBOV GP containing or lacking the mucin-like domain (MLD). The literature more completely describes monoclonal antibodies against the GP core, as MLD-directed mAbs rarely neutralize, and mAbs against the more-conserved GP core or glycan cap are more likely to be cross-reactive [14,42]. The unique reactivity of some individuals to only EBOV GPe and not to EBOV GPΔMuc observed here suggests that these GPe^+^ individuals may express MLD-specific antibodies. Other individuals reacted only to mucin-deleted GP, and not to mucin-containing GPe. Of these EBOV GPΔmuc^+^/GPe^-^ individuals, one (HCW354) also reacted with mucin-deleted BDBV and SUDV GPs, suggesting that their cross-reactive antibody response is against the more-conserved EBOV GP core, rather than the MLD. None of these five EBOV GPΔmuc^+^/GPe^-^ individuals would have been detected in prior serology assays that use only mucin-containing GP. The lack of consistently coupled EBOV GPΔMuc and GPe reactivity suggests individuality in the immune response, and that using just one form of the antigen in serological testing may limit detection of prior infections. Using both forms of the antigen would offer more complete detection.

There are some limitations with this study resulting from performing data acquisition in the DRC. The structural integrity of the antigens used in this study, particularly EBOV GPΔMuc, is sensitive to fluctuations in temperature. During sample analysis, temperature fluctuations due to power outages were recorded. We did observe differences in the EC_50_ of Adimab-15878 when the assay was performed post-outage, and adjusted for interplate variation in our analysis. Loss of cold chain however, may have led to loss of high affinity binding and fewer positive detections than if stable infrastructure had been present.

Further, due to lack of access to sufficient equipment and biosafety containment in the lab in Kinshasa, we could not conduct neutralization studies using authentic virus or pseudovirus. As such, we could not determine the neutralization capacity of the sera tested in this study. However, studies by Adaken et al. and Diallo et al. show that levels of binding antibody correlate with neutralization and/or protection from disease [43,44]. Further, we could not test for antibodies against Marburg or Ravn viruses due to a lack of an adequate positive control at the time of testing. Future testing of these samples against Marburg and/or Ravn GP could show if cross-reactivity amongst ebolaviruses extends to reactivity against filoviruses as a whole.

In addition to biochemical limitations, our study may have been affected by other sources of bias. We employed a convenience sample, which may have limited the generalizability of our findings or induced selection bias. Furthermore, our estimates in Table 4 could be affected by uncontrolled confounding. Additionally, data was collected through self-report, which may be subject to bias due to limitations of recall and translation errors. However, substantial effort was undertaken to reduce information bias due to translation errors. Local staff were hired to administer questionnaires to conserve information in each translation from local languages to English and vice versa.

Ultimately, our broad, pan-ebolavirus serological study offers insight into a population’s humoral immune response against ebolaviruses. Our findings highlight the depth and breadth of the IgG response against multiple ebolavirus GPs, including those from ebolavirus species that have never been previously reported in the area. Interestingly, no strong pairwise antigen response correlations or predictors of cross-reactivity were identified to give insight into the observed cross-reactivity in our sample. We demonstrate that HCWs, particularly those who may not receive workplace infection-control training or sufficient PPE for patient interaction, may have a higher likelihood of ebolavirus exposure. Furthermore, our results add to the growing body of evidence indicating potential mild or asymptomatic ebolavirus infection. Future applications of this assay include rapid and consistent determination of whether an individual has mounted a strong IgG response to multiple ebolaviruses and screening for development of therapeutic antibodies.

## Supporting information

S1 Table EC_50_-corrected raw ELISA data. (XLSX)
